# Optimization of the process of seed extraction from the *Larix decidua* Mill. cones including evaluation of seed quantity and quality

**DOI:** 10.1038/s41598-022-22942-2

**Published:** 2022-10-29

**Authors:** Ewa Tulska, Monika Aniszewska, Witold Zychowicz

**Affiliations:** Department of Biosystems Engineering, Institute of Mechanical Engineering, Warsaw University of Life Sciences–SGGW, Nowoursynowska, 164, 02-787 Warsaw, Poland

**Keywords:** Mechanical engineering, Forest ecology, Forestry, Scientific data

## Abstract

The objective of this study was to determine the number of stages of cone drying and immersion that yield the maximum number of high quality seeds. Nine variants of the process were conducted; they differed in terms of dwell time in the drying chamber and water immersion time. Each extraction variant consisted of five drying steps (lasting 10, 8 or 6 h) and four immersion steps (5, 10 or 15 min). Each drying step was followed by cone shaking in a purpose-made laboratory drum. The process variants were evaluated and compared in terms of cone moisture content as well as the dynamics of seed yield and the quality of seeds obtained in the various steps. The seed yield coefficient, α, and the cone mass yield coefficient, β, were calculated. The studied process of seed extraction can be described using the Lewis empirical model for the second stage of drying with the *b* coefficient ranging from 0.34 to 0.60. Relatively higher initial and final moisture content was found for cones immersed for 15 min (more than 0.45 kg_water_·kg_d.w._^−1^), while the lowest moisture content was found for those immersed for 5 min (less than 0.4 kg_water_·kg_d.w._^−1^). The highest seed yield at the first and second steps was obtained in the 8 h_10 min variant (53% and 32%, respectively). In all five-step variants, the mean cone yield amounted to 65% of total seeds in the cones; seeds obtained from all variants were classified in quality class I. The procedure recommended for commercial seed extraction facilities consists of three 8 h drying steps and two 10 min immersion steps, with cone shaking in a drum to maximize seed yield. A shorter cone extraction process maintaining an acceptable level of seed extraction may reduce energy consumption by nearly 50%.

## Introduction

It is more difficult to extract seeds from the cones of the European and Polish larch^1,2^ than from other conifer species^3,4^. This is due to the specific anatomical and morphological structure of larch cone scales^5,6^. As compared to pine and spruce, the scales of larch cones are thinner, smaller, and more rounded^4^. They can be used to determine the exact taxonomic species of larch trees^7^. In a typical European larch, the scales are relatively broad and taper towards the tip; the top margin is oval and sometimes crenate^4^. According to the literature, scales are made of layers of cells with different wall thicknesses. Moisture is evaporated most readily via cells located on the external side, due to which scales are deflected away from the rachis in their middle and top regions. Dry scales are brittle, and upon breaking they lose elasticity and do not deflect again easily^8^. The specific mechanisms of scale opening and closing are associated with tree survival strategy and evolution, which allowed coniferous species to release seeds at greater distances on dry sunny days^9,10^.

The process of seed extraction is used for controlled seed production in silvicultural extractories^11^. Seeds obtained in this way are stored short-term (for same-season sowing)^12^ or long-term^13^, e.g., ^14^ in seed banks or in nurseries (to be sowed in the open or in containers–single-seed sowing)^15,16^. The right temperature and moisture conditions of seed extraction guarantee the production of high-quality seeds ^3^.

The issue of standardization of seed extraction from larch cones (*Larix*) was predominantly studied at the time of afforestation of war-affected areas from the 1950s^17^ to the 1980s^18–22^. The current literature on seed extraction largely focuses on pine cones due to the high demand for pine seeds^23–25^. The control of energy use in the process of seed extraction is important to ensure sustainable development while promoting the conservation of natural resources. Energy consumption has become a significant influence on every country’s economic progress^26^. Manufacturers have two basic options of reducing reliance on fossil fuels and at the same time diminishing the environmental impact of their activity: the use of renewable energy systems or a decrease in energy consumption^27^. The reduction of demand for energy can be further subdivided into three options: a reduction in total activity (e.g.^28^); improved energy management (e.g.^29^); and the recovery and reuse of waste energy (e.g.^30^).

The genus *Larix* is used for fast-growth plantations in Poland^31^, with the species being dominant in China and Mongolia^32^, as well as Canada^33^. Comminuted larch bark is an important source of biomass and can be used to manufacture upcycled products (Tudor et al.^34^). For example, thin panels made from comminuted bark can replace cork in flooring applications^35^. Additionally, the pulp and paper industry produces many by-products, some of which are used to produce electricity^36^. The pursuit of the full and effective use of biological material (larch trees) is the reason why research efforts should continue optimizing the process of extracting seeds from the cones of this species, which has been known in Poland for over a century^37^. There are gaps in knowledge about the number of sequential drying and immersion steps and their duration to ensure the maximum effectiveness of the seed extraction process.

Given the difficulties associated with seed extraction from larch cones, two methods are predominantly used in practice: thermal and thermo-mechanical^3^. The former involves cone immersion or spraying in addition to drying, which significantly extends the time of seed extraction, even up to 60 h^4^. The time the cones spend in the dryer can take up to half the time of the entire process of seed extraction, which makes drying the most energy-consuming part of the process.

In the thermo-mechanical method, moisture content in the cones is reduced to about 8–10%^38^, making the scales so brittle that they break off the rachis and release the seeds more easily^8^. Additionally, mechanical shaking or crushing results in a greater seed yield, but during such a procedure the seed coat may be damaged by abrasive elements^3^. Following such a process, the seeds should be separated from the mixture of dust and cone remains, which presents a difficulty in terms of sorting empty and full larch seeds, as their weight is similar^3,4^. It should be noted that the proportion of empty seeds in larch cones may reach up to 70%^21^. Such procedures also cause high levels of dust in the seed extraction facility, which is hazardous and requires additional precautions on the part of the operators.

Polish extraction facilities increasingly use the thermal method which involves cone moistening in multi-step larch seed extraction^3^. A two-step extraction process in extractor cabinets with variable drying temperature for pine and spruce cones^17^ has been adopted for larch cones. This leads to high-quality seeds with high germination energy and capacity^8,39^. In practice, a three-step process with a significant reduction in moisture content is used most often^40^. To attain maximum extraction yield, larch cones should be alternately dried and moistened^19^. However, cones should not be immersed for too long, as it could have an adverse effect on the viability of the obtained seeds. Furthermore, after several hours in water, the seeds swell^8^ and must be sown shortly after extraction. Practitioners use two types of cone moistening: immersion (e.g., seed extractory in Lasowice Małe; Kluczbork Forest District) and spraying (e.g., seed extractory in Czarna Białostocka; Czarna Białostocka Forest District).

The research problem arises from the absence of detailed information concerning the commercial extraction of seeds from larch cones or the efficiency of the thermal method (with immersion) applied for that purpose. It is not known how many drying and immersion steps should be used to ensure the right drying/moisture parameters to obtain the greatest possible quantity of seeds without compromising their quality. Furthermore, the duration of such steps should be investigated to make sure that the process is economically and energetically sound.

It is possible that there exists a certain threshold of cone moisture content at which seeds are released. It is likely that after the cone drying and immersion steps, the next drying step will be facilitated by the phenomenon of drying contraction^41^. Thus, it is predicted that during the subsequent immersion step the cones will be able to absorb more water.

Another objective of the paper is to analyze changes in cone moisture content during a five-step extraction process with varied seed drying and immersion times, to develop a mathematical model of heat and mass exchange during extraction, to evaluate the dynamics of extraction in the studied process variants, and to assess the quality of the obtained seeds.

## Materials and methods

### Material provenance and characteristics

The laboratory study involved European larch cones collected from the seed orchard at the beginning of December 2019 in the Grabowiec Nursery, division 282 k, in Bielsk Podlaski municipality, Podlaskie Province (52° 41′ 0 N, 23° 60′ E), by the Czarna Białostocka seed extraction facility. Plant study was carried out in accordance with the regulations in force in the State Forests in Poland. Permission was obtained from the municipality of Bielsk Podlaski, Podlaskie province, to use the collected larch cones for research. The national number in the register of suppliers of primary forestry material from which the cones were obtained was MP/3/41001/05, and the certificate of provenance for the cones was MR/60849/20/PL (based on the Polish Act of June 7, 2001 on forest reproductive material (Journal of Laws of 2015, item 1092)). The collected cones were transported to the laboratory of the Department of Biosystems Engineering, Warsaw University of Life Sciences, divided into batches and stored in an LKexv 3600 laboratory refrigerator (Liebherr, Bulle, Switzerland) at + 2 ± 1 °C prior to the study. All cones had their height (*h*) and diameter (*d*) measured using an electronic sliding caliper (model 677,256 from Silverline Tools, Yeovil, Great Britain) with an accuracy of ± 0.1 mm, and their initial weight was determined using a WPS210S laboratory balance (Radwag, Radom, Poland) with an accuracy of ± 0.001 g.

### Seed extraction process: its course and empirical model

The cones were divided into ten batches with 32 cones per batch; nine batches were designated for testing the extraction process and one constituted controls not subjected to any treatment. The controls were placed in individual containers without drying, soaking or shaking. They were kept at room temperature in open containers. The seeds that were naturally released from them were collected and transferred to a germinator.

The nine studied seed extraction variants differed in terms of drying and immersion times. Each variant of the procedure consisted of five drying steps of 10, 8 or 6 h, five steps of shaking in a drum for 30 min, four immersion steps of 5, 10 or 15 min, and four soaking steps of 12, 14 or 16 h. A stage is defined as one single cycle of drying, shaking and optionally immersion and soaking the cones. Variants are the names of entire processes, each of which begins with placing the cones in a dryer and ends with an evaluation of the quality of the seeds obtained in the process.

**Figure 1:**
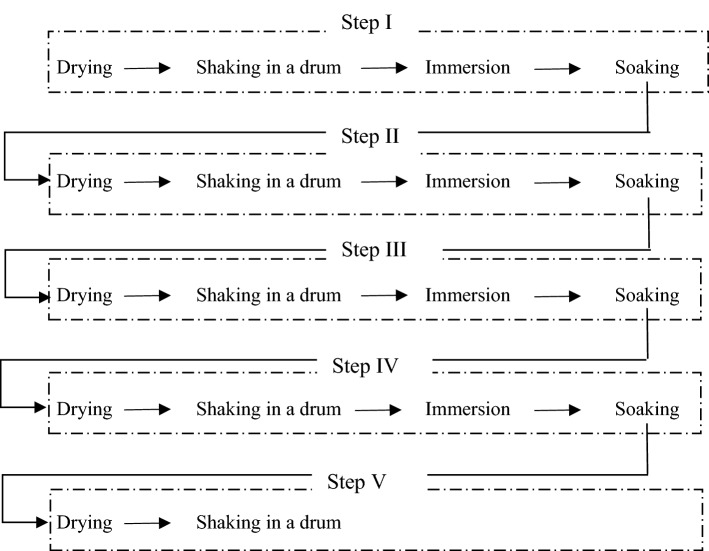
Scheme of the seed extraction process: (**a**) drying; (**b**) shaking in a drum; (**c**) immersion; (**d**) soaking.

Effective extraction time was defined as the sum of all drying, immersion and shaking times during the entire procedure. In turn, total process time also included cone soaking after steps 1, 2, 3, and 4 (Fig. 1, Table 1).

**Table 1:** Scope of laboratory studies of seed extraction.

Nr	Variant	Total drying time in the chamber [h]	Total immersion time [min]	Total shaking time [min]	Total soaking time [h]	Effective extraction time	Total process time [h]
1	10 h_5 min	50	20	150	~ 48	50 h + 20 min + 150 min	~ 101
2	10 h_10 min	50	40	150		50 h + 40 min + 150 min	
3	10 h_15 min	50	60	150		50 h + 60 min + 150 min	
4	8 h_5 min	40	20	150	~ 56	40 h + 20 min + 150 min	~ 99
5	8 h_10 min	40	40	150		40 h + 40 min + 150 min	
6	8 h_15 min	40	60	150		40 h + 60 min + 150 min	
7	6 h_5 min	30	20	150	~ 64	30 h + 20 min + 150 min	~ 97
8	6 h_10 min	30	40	150		30 h + 40 min + 150 min	
9	6 h_15 min	30	60	150		30 h + 60 min + 150 min	

After removing from the refrigerator, the cones were acclimatized at room temperature (20–22 °C) for about 1 h at a relative air humidity of 40%. Then, after measuring the size and weight parameters, a batch of 32 cones was placed on perforated shelves in a Heraeus UT612 convection dryer with air circulation (Kendro Laboratory Products GmbH, Hanau, Germany). At the beginning of each drying step, the temperature in the dryer was 35 °C for the first 2 h, and then it was increased to 50 °C until the end of the process to prevent the seeds from thermal damage. During the process, the cones were removed from the dryer every 2 h to weigh them and separate the seeds that had been released. The measurement time was about 5 min.

After each drying step, individual cones were put in tightly sealed P17 geotextile bags, and then placed in a drum and shaken for 30 min. The drum rotated at 30 rpm and was tilted at 30° from the horizontal (patent claim no. UP P.437143 [WIPO ST 10/C PL437143]).

After shaking, the seeds were removed from each bag, and the number and weight of the seeds were recorded. Subsequently, the cones were immersed in plastic containers half-filled with distilled water at 24 °C (± 1 °C) for 5, 10 or 15 min, depending on the test variant. During immersion time, the cones were covered by water. After immersion, the cones were taken out of the containers and placed on a cotton fabric at a controlled temperature of 22 °C for 12, 14, or 16 h for variants including 10, 8 or 6 h drying steps, respectively. After the appropriate time of drying, shaking, and then soaking and soaking—the first stage of the process is complete.

During the second and subsequent drying steps, the cones were weighed prior to placing them in the dryer; the rest of the procedure was exactly the same as before. In each variant, after the fifth—last step, neither immersion nor soaking was performed (only drying and shaking of cones).

The temperature and humidity in the dryer and in the laboratory were monitored with a FTH 100 m (Geo FENNEL, Kassel Germany) with an accuracy of 0.01 °C and 0.01%, respectively.

Following the extraction process, the cones were dried at 105 °C (± 1 °C) in the dryer to constant weight. Dry weight measurements made it possible to calculate the initial and final moisture content as well as determine instantaneous moisture content in the cones throughout the process.

It was assumed that changes in cone moisture content during the five-step processes can be described according to the Lewis empirical model for the second stage of drying ( ^42,43^). The model was adapted to the extraction conditions by determining the values of the coefficients for each of the five drying steps taking into consideration variable temperature in the drying chamber and other determinants. The general equation describing moisture content changes in cones for one step has the following form^44^:

1 $$u = \left( {u_{0} - u_{k} } \right) \cdot e ^{{\left( { - b \cdot \tau_{i} } \right)}} + u_{k}$$

where *u*–instantaneous cone moisture content during the extraction process ${[\mathrm{kg}}_{\mathrm{water}}\cdot {\mathrm{kg}}_{\mathrm{d}.\mathrm{w}.}^{-1}]$, *u*_*0*_– initial cone moisture content in a given step ${[\mathrm{kg}}_{\mathrm{water}}\cdot {\mathrm{kg}}_{\mathrm{d}.\mathrm{w}.}^{-1}]$, *u*_*k*_–final cone moisture content in a given step ${[\mathrm{kg}}_{\mathrm{water}}\cdot {\mathrm{kg}}_{\mathrm{d}.\mathrm{w}.}^{-1}]$, *b*–coefficient characterizing moisture content change [h^−1^], $\tau$_i_–time [h], *e*–the base of the natural logarithm.

The value of coefficient *b* given in the literature^44^ is constant and reflects changes in the moisture content of cones dried at a constant temperature^40^.

In addition, the seed extraction rate was calculated from the formula below:

2 $$\frac{du}{{d\tau_{i} }} = - b \cdot \left( {u_{0} - u_{k} } \right) \cdot {\text{e }}^{{\left( { - b \cdot \tau_{i} } \right)}}$$

Formulas 1 and 2 were used to describe the course of seed extraction in successive steps of the studied nine variants.

### Seed extraction dynamics

The dynamics of seed extraction was assessed by calculating the cumulative number and weight of the seeds released in the process using the following formula for the seed yield coefficient, *α* (3), and the cone mass yield coefficient, *β* (4):

3 $$\alpha = \frac{{l_{n} }}{{l_{w} }}$$

where *l*_*n*_–number of extracted seeds, *l*_*w*_–total number of seeds;

4 $$\beta = \frac{{m_{n} }}{{m_{0} }}$$

where *m*_*n*_– weight of extracted seeds [g], *m*_*0*_– initial weight of the cone [g].

After the extraction process was completed, the number of open scales capable of producing two seeds was analyzed, as they are structural elements determining the course of seed extraction.

### Seed viability assessment

The seeds obtained from each step of the studied process variants were manually dewinged, cleaned and transferred to a Jacobsen germinator (Laborset, Łódź, Poland) to assess seed viability according to the Polish Seed Evaluation System^45,46^. Larch seeds were placed on filter paper in the germinator in three replicates, with 100 samples per seed batch. The 24 h germination cycle included 8 h of illumination, with the water temperature being maintained at 24 °C. Temperature was controlled using a Termo-Stab RBS1 controller (Termo-Stab, Warsaw, Poland) with an accuracy of ± 1 °C. The applied exposure timer was a Grasslin Talento 371 device (Grässlin GmbH, St. Georgen, Germany). The light source consisted of two Tungsram 36 W-F74 Daylight fluorescent tubes. Germination energy was measured after seven days, and germination capacity after 28 days of the experiment. These results were used to determine the seed quality class pursuant to the standard PN-R-65700. First class larch seeds should germinate in 40–60% of the cases.

### Statistical analysis

Statistical analyses were performed using the Statistica v. 13 (2010) software. The normal distribution of cone size and weight parameters was assessed using the Shapiro–Wilk test. Differences in mean size and weight parameters were assessed by ANOVA *F* and Duncan’s test. The homogeneity of variance was also tested using the HSD Tukey test. All analyses were performed at a statistical significance level of 0.05.

## Results

### Cone characteristics: the entire set and individual variants

Cones used in all the test variants did not differ from each other in terms of height (coefficient of variance in the Student *t*-test–*F* = 1.33 at *p* = 0.23), diameter (*F* = 1.77 at *p* = 0.08), or initial weight (*F* = 0.86 at *p* = 0.55). Analysis of variance revealed a significant difference for cone humidity (*F* = 2.52 at *p* ˂ 0.05).

Cone parameters such as height, diameter and initial weight are factors that can determine the course of the extraction process. Therefore, the relationship between diameter and height for all cones used in the study was described using a linear regression equation $(y=0.2794x+8.3195)$, which means that cone diameter increased by 0.28 mm per 1 mm of cone height, $(R=0.778>0.104-{R}_{kr})$.

The initial weight of cones may be associated with their harvest time or storage conditions. A linear regression equation was also used to describe the relationship between the height and initial weight of the examined cones (y = 0.238x–3.918), which means that initial weight increased on average by 0.238 g per 1 mm of height, (*R* = 0.795 > 0.104).

Table 2 shows means with standard deviations, the minimum and maximum values of the measured parameters, the range of variance, the coefficient of variation and the standard error for the entire set of studied cones and seeds. The Shapiro–Wilk test showed that the examined characteristics had a normal distribution.

**Table 2:** Cone and seed parameters for the entire study set.

Parameter	Means (± standard deviation)	Min	Max	Range of variance	Coefficient of variation	Standard error
Height *h* [mm]	33.8 ± 3.4	21.4	44.1	22.7	10.1	0.2
Diameter *d* [mm]	17.8 ± 1.6	12.5	24.3	11.8	8.8	0.1
Initial weight *m*_*0*_ [g]	4.144 ± 1.019	2.137	9.111	6.974	24.585	0.060
Initial moisture *W* [%]	40.4 ± 4.5	27.6	57.1	29.5	11.2	0.3
Number of scales *l*_*scales*_	48 ± 6	33	70	37	13	0.36
Number of extracted seeds *l*_*n*_	36 ± 18	1	96	95	49	1
Total number of seeds *l*_*w*_	52 ± 19	5	97	92	37	1
Weight of extracted seeds *m*_*n*_ [g]	0.193 ± 0.109	0.000	0.651	0.651	56.541	0.006

The cones used in the study had a height of 21.4–44.1 mm and a diameter of 12.5–24.3 mm. The mean height of a cone was 33.8 (± 3.4) mm and the mean diameter was 17.8 (± 1.6) mm. The initial weight of cones ranged from 2.137 to 9.111 g, with a mean of 4.144 (± 1.019) g. The initial moisture content of cones was from 27.6 to 57.1%, with a mean of 40.4 (± 4.5)%. Analysis was performed for individual extraction variants. The mean values of cone height *h*, diameter *d*, initial weight *m*_*01*_, and moisture content *W* were calculated (Table 3).

**Table 3:** Mean parameter values and standard deviations for the nine process variants.

No	Variant	Height *h* [mm]	Diameter *d* [mm]	Initial weight *m*_*01*_ [g]	Moisture content *W* [%]
1	10 h_5 min	33.7^a^ ± 3.3	17.7^a,b^ ± 1.4	3.945^a^ ± 1.109	40.22^a,b^ ± 3.57
2	10 h_10 min	32.3^a^ ± 3.5	17.4^a^ ± 1.3	3.949^a^ ± 0.961	41.48^a^ ± 4.33
3	10 h_15 min	33.3^a^ ± 3.6	17.5^a,b^ ± 1.5	4.073^a^ ± 0.989	41.56^a^ ± 3.64
4	8 h_5 min	33.8^a^ ± 3.2	17.6^a,b^ ± 1.3	4.134^a^ ± 1.004	41.03^a,b^ ± 5.18
5	8 h_10 min	34.0^a^ ± 3.7	17.6^a,b^ ± 1.8	4.320^a^ ± 0.863	41.05^a,b^ ± 5.38
6	8 h_15 min	34.2^a^ ± 3.2	17.6^a,b^ ± 1.8	4.459^a^ ± 1.223	41.41^a,b^ ± 3.37
7	6 h_5 min	34.5^a^ ± 3.1	18.6^b^ ± 1.6	4.152^a^ ± 1.072	38.00^b^ ± 5.21
8	6 h_10 min	33.9^a^ ± 3.7	18.0^a,b^ ± 1.7	4.208^a^ ± 0.881	38.98^a,b^ ± 4.22
9	6 h_15 min	34.6^a^ ± 3.3	18.0^a,b^ ± 1.4	4.144^a^ ± 1.019	39.83^a,b^ ± 4.41

*a, b – homogeneous groups.*

The HSD Tukey test revealed one homogeneous group for cone height encompassing all variants and two homogeneous groups for diameter. The first group consisted of all variants except 7, and the second group included all variants except 2. One homogeneous group was obtained for initial weight. Two homogeneous groups were found for moisture content, one consisting of all variants except 7, and the other one containing variants 1, 4, 5, 6, 7, 8, and 9.

### Seed extraction results for the studied steps

#### Seed extraction conditions and time

The change in cone weight in each step of the extraction process depended on its duration, temperature and humidity conditions in the extraction cabinet, as well as on the initial moisture content of the cones.

Humidity inside the drying chamber decreased to an average of 30% after 2 h of the process in each step as a result of increasing temperature. Over the subsequent 4 h of the process, after increasing the temperature, the humidity inside the chamber declined significantly, and then (over 2 and 4 h) it decreased further only slightly, stabilizing at approx. 5% for the 10 h variants, 6% for the 8 h variants, and 8% for 6 h variants on average.

#### Moisture content changes in cones during the seed extraction process

The initial moisture content (*u*_*01*_) of the studied cones was much greater than 0.20 ${\mathrm{kg}}_{\mathrm{water}}\cdot {\mathrm{kg}}_{\mathrm{d}.\mathrm{w}.}^{-1}$, which means that special care must be taken during seed extraction, which should be conducted at a temperature of up to 50 °C^8^.

The relatively high moisture content of the cones could be attributed to the absence of preliminary drying in airy storage places prior to seed extraction (which is typically the case in commercial practice) and the early date of cone harvest, at the beginning of the extraction season. The initial (*u*_*0x*_) and final (*u*_*kx*_) moisture content of cones used in each process variant is given with standard deviation in Table 4.

**Table 4:** Initial and final moisture content of cones used in each process variant.

No	Variant	Moisture content of cones ${[\mathrm{kg}}_{\mathrm{water}}\cdot {\mathrm{kg}}_{\mathrm{d}.\mathrm{w}.}^{-1}]$									
		*u*_*01*_	*u*_*k1*_	*u*_*02*_	*u*_*k2*_	*u*_*03*_	*u*_*k3*_	*u*_*04*_	*u*_*k4*_	*u*_*05*_	*u*_*k5*_
1	10 h_5 min	0.402^a,b^ ± 0.036	0.131^a^ ± 0.022	0.408^a^ ± 0.054	0.108^a^ ± 0.022	0.413^a,b^ ± 0.053	0.085^a^ ± 0.013	0.414^b^ ± 0.057	0.069^a^ ± 0.008	0.415^a^ ± 0.053	0.071^a,b^ ± 0.004
2	10 h_10 min	0.415^a^ ± 0.043	0.130^a^ ± 0.021	0.422^a^ ± 0.053	0.108^a^ ± 0.018	0.424^a,b^ ± 0.054	0.087^a^ ± 0.013	0.426^a,b^ ± 0.051	0.070^a^ ± 0.008	0.429^a,b^ ± 0.055	0.070^a,b^ ± 0.004
3	10 h_15 min	0.416^a^ ± 0.036	0.129^a^ ± 0.024	0.425^a^ ± 0.060	0.110^a^ ± 0.019	0.450^b^ ± 0.055	0.086^a^ ± 0.012	0.466^b^ ± 0.080	0.071^a^ ± 0.007	0.468^b^ ± 0.066	0.071^a,b^ ± 0.004
4	8 h_5 min	0.410^a,b^ ± 0.052	0.126^a^ ± 0.026	0.406^a^ ± 0.061	0.090^b^ ± 0.017	0.421^a,b^ ± 0.061	0.081^a^ ± 0.011	0.422^a,b^ ± 0.081	0.072^a^ ± 0.015	0.424^a,b^ ± 0.084	0.064^a^ ± 0.011
5	8 h_10 min	0.411^a,b^ ± 0.054	0.126^a^ ± 0.037	0.409^a^ ± 0.046	0.090^b^ ± 0.024	0.433^a,b^ ± 0.048	0.080^a^ ± 0.010	0.430^a,b^ ± 0.053	0.071^a^ ± 0.010	0.444^a,b^ ± 0.051	0.065^a^ ± 0.009
6	8 h_15 min	0.414^a,b^ ± 0.034	0.132^a^ ± 0.027	0.429^a^ ± 0.059	0.106^a^ ± 0.023	0.438^a,b^ ± 0.054	0.086^a^ ± 0.013	0.449^a,b^ ± 0.059	0.070^a^ ± 0.009	0.452^a,b^ ± 0.067	0.064^a^ ± 0.005
7	6 h_5 min	0.380^b^ ± 0.052	0.121^a^ ± 0.022	0.404^a^ ± 0.059	0.106^a^ ± 0.022	0.372^c^ ± 0.045	0.090^a,b^ ± 0.018	0.393^a^ ± 0.068	0.077^b^ ± 0.022	0.398^a^ ± 0.076	0.077^b,c^ ± 0.026
8	6 h_10 min	0.390^a,b^ ± 0.042	0.139^a^ ± 0.024	0.405^a^ ± 0.035	0.114^a^ ± 0.019	0.403^a,c^ ± 0.038	0.101^b,c^ ± 0.020	0.403^b^ ± 0.058	0.091^b,c^ ± 0.011	0.411^a^ ± 0.046	0.081^c^ ± 0.017
9	6 h_15 min	0.398^a,b^ ± 0.044	0.129^a^ ± 0.038	0.420^a^ ± 0.048	0.117^a^ ± 0.018	0.423^a,b^ ± 0.058	0.105^c^ ± 0.016	0.428^a,b^ ± 0.067	0.100^c^ ± 0.015	0.436^a,b^ ± 0.066	0.093^d^ ± 0.018

*a, b, c, d – homogeneous groups.*

The initial moisture content of cones (*u*_*0x*_) in most variants increased with each extraction step due to immersion. In most variants, the final moisture content (*u*_*kx*_) was the highest in the first extraction step and decreased or remained at the same level with each subsequent step.

The mean initial moisture content for the three process variants with 10 h of drying was 0.411 ${\mathrm{kg}}_{\mathrm{water}}\cdot {\mathrm{kg}}_{\mathrm{d}.\mathrm{w}.}^{-1}$. After 10 h of drying, the mean moisture content decreased to 0.130 ${\mathrm{kg}}_{\mathrm{water}}\cdot {\mathrm{kg}}_{\mathrm{d}.\mathrm{w}.}^{-1}$. The mean initial moisture content in the fifth extraction step was 0.437 ${\mathrm{kg}}_{\mathrm{water}}\cdot {\mathrm{kg}}_{\mathrm{d}.\mathrm{w}.}^{-1}$, and the final moisture content in that step was 0.071 ${\mathrm{kg}}_{\mathrm{water}}\cdot {\mathrm{kg}}_{\mathrm{d}.\mathrm{w}.}^{-1}$ . Cones dried for 10 h reached on average 7% moisture content after extraction steps 4 and 5.

The mean initial moisture content for the three process variants with 8 h of drying was 0.412 ${\mathrm{kg}}_{\mathrm{water}}\cdot {\mathrm{kg}}_{\mathrm{d}.\mathrm{w}.}^{-1}$. After 8 h of drying, the mean moisture content decreased to 0.128 ${\mathrm{kg}}_{\mathrm{water}}\cdot {\mathrm{kg}}_{\mathrm{d}.\mathrm{w}.}^{-1}$ . The mean initial moisture content in the fifth extraction step was 0.440 ${\mathrm{kg}}_{\mathrm{water}}\cdot {\mathrm{kg}}_{\mathrm{d}.\mathrm{w}.}^{-1}$, and the final moisture content in that step was 0.064 ${\mathrm{kg}}_{\mathrm{water}}\cdot {\mathrm{kg}}_{\mathrm{d}.\mathrm{w}.}^{-1}$ . Cones dried for 8 h reached on average 7.1% moisture content after extraction step IV and 6.4% after step V.

The mean initial moisture content for the three process variants with 6 h of drying was 0.389 ${\mathrm{kg}}_{\mathrm{water}}\cdot {\mathrm{kg}}_{\mathrm{d}.\mathrm{w}.}^{-1}$. After 6 h of drying, the mean moisture content decreased to 0.129 ${\mathrm{kg}}_{\mathrm{water}}\cdot {\mathrm{kg}}_{\mathrm{d}.\mathrm{w}.}^{-1}$ . The mean initial moisture content in the fifth extraction step was 0.415 ${\mathrm{kg}}_{\mathrm{water}}\cdot {\mathrm{kg}}_{\mathrm{d}.\mathrm{w}.}^{-1}$, and the final moisture content in that step was 0.084 ${\mathrm{kg}}_{\mathrm{water}}\cdot {\mathrm{kg}}_{\mathrm{d}.\mathrm{w}.}^{-1}$ . Cones dried for 6 h reached on average 8.9% moisture content after extraction step IV and 8.4% moisture content after step V, which means that their final moisture content was higher than that of cones dried for 8 h and 10 h.

The cones with the longest immersion time (15 min) were characterized by the highest initial moisture content in each extraction step as compared to the other two variants (immersion of 5 min and 10 min) with the same drying time. The final moisture content in a given extraction step differed between cones with different immersion times. Cones with an immersion time of 15 min were characterized by the highest final moisture content in individual extraction steps, and those with 5 min immersion revealed the lowest final moisture content.

The Tukey HSD test revealed homogeneous groups in terms of initial moisture content (*u*_*01*_*, u*_*02*_*, u*_*03*_*, u*_*04*_*, u*_*05*_) and final moisture content (*u*_*k1*_*, u*_*k2*_*, u*_*k3*_*, u*_*k4*_*, u*_*k5*_) in each step, as shown in Table 4. For instance, four homogeneous groups were found for the final moisture content after extraction step V (*u*_*k5*_): the first one consisted of all variants except for 7, 8, and 9, the second one included variants 1, 2, 3, and 7, the third one comprised of variants 7 and 8, while the fourth one was constituted by variant 9 alone.

Using Eq. (1), changes in moisture content were described for each of the tested cones over all five steps of each variant. The equation included the initial and final values of moisture content and the *b* coefficient for individual cones. The average values of the *b* coefficient and standard deviations for each extraction step are presented in Table 5 for individual extraction variants.

**Table 5:** Mean values of the *b* coefficient and standard deviations for the five steps of the studied process variants.

No	Variant	Values of *b* coefficient				
		*b*_*1*_	*b*_*2*_	*b*_*3*_	*b*_*4*_	*b*_*5*_
1	10 h_5 min	0.36^a^ ± 0.04	0.41^a^ ± 0.04	0.48^a^ ± 0.05	0.46^a^ ± 0.05	0.45^a^ ± 0.05
2	10 h_10 min	0.35^a^ ± 0.03	0.43^a^ ± 0.04	0.51^a,b^ ± 0.06	0.48^a^ ± 0.05	0.46^a^ ± 0.05
3	10 h_15 min	0.34^a^ ± 0.04	0.42^a^ ± 0.04	0.50^a,b^ ± 0.05	0.45^a^ ± 0.04	0.50^c^ ± 0.05
4	8 h_5 min	0.45^d^ ± 0.07	0.52^d^ ± 0.09	0.50^a,b^ ± 0.05	0.46^a^ ± 0.05	0.46^a^ ± 0.05
5	8 h_10 min	0.40^c^ ± 0.07	0.46^c^ ± 0.08	0.47^a^ ± 0.05	0.46^a^ ± 0.05	0.46^a^ ± 0.04
6	8 h_15 min	0.43^c,d^ ± 0.08	0.47^c^ ± 0.04	0.53^b,c^ ± 0.06	0.57^b^ ± 0.06	0.60^d^ ± 0.07
7	6 h_5 min	0.53^b^ ± 0.05	0.55^b^ ± 0.03	0.55^c,d^ ± 0.03	0.55^b^ ± 0.03	0.54^b,c^ ± 0.03
8	6 h_10 min	0.55^b^ ± 0.06	0.58^b^ ± 0.06	0.57^d^ ± 0.05	0.58^b^ ± 0.06	0.57^b,d^ ± 0.05
9	6 h_15 min	0.53^b^ ± 0.04	0.55^b^ ± 0.04	0.55^c,d^ ± 0.03	0.57^b^ ± 0.04	0.55^b^ ± 0.03

*a, b, c, d – homogeneous groups.*

The lowest value of the *b* coefficient was recorded for the first step of the 10h_15min variant (*b*_*1*_ = 0.34), while the highest value was obtained for the fifth step of the 8 h_15 min variant (*b*_*5*_ = 0.60). In the process variants involving 10 and 8 h of drying , the *b* coefficient increased with each extraction step until the third one; in the fourth step it slightly decreased and in the fifth step it remained constant. In the variants with 6 h of drying the *b* coefficient almost peaked in the second extraction step and remained at a similar level until the fifth step. In the first steps of the variants with 6 h of drying, the mean value of the *b* coefficient was 0.54 and did not differ significantly from the coefficients obtained during the other steps. It was noted that in the 8 h_15 min variant, the *b* coefficients increased over successive steps.

**Figure 2:**
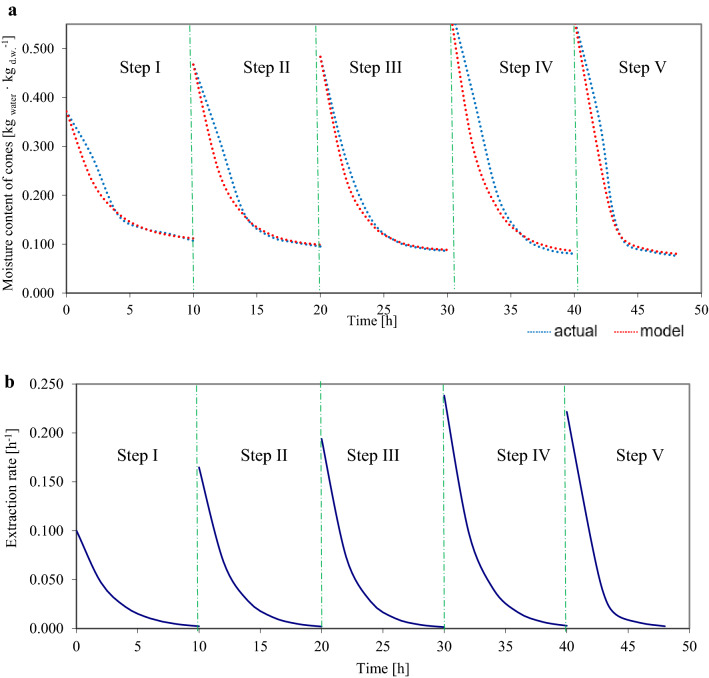
Diagrams: (**a**) actual and predicted changes in cone moisture content, (**b**) extraction rate in five extraction steps for larch cone no. 32 in the 10 h_15 min variant throughout effective extraction.

**Figure 3:**
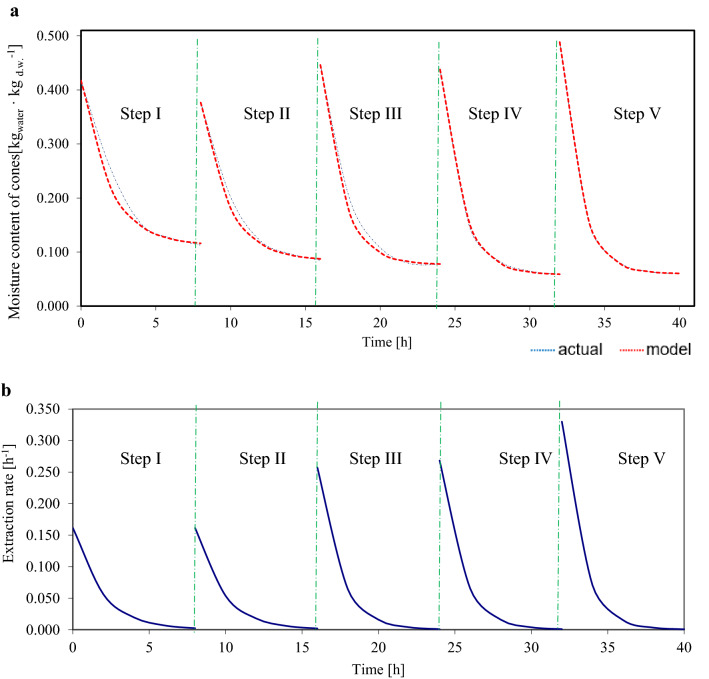
Diagrams: (**a**) actual and predicted changes in cone moisture content, (**b**) extraction rate in five extraction steps for larch cone no. 17 in the 8 h_15 min variant throughout effective extraction.

Figures 2–3 show examples of curves of actual and model changes in moisture content and the rate of extraction for sample cones, one each for variants 10 h_15 min and 8 h_15 min.

Equations for changes in moisture content and extraction rate in consecutive extraction steps are given below for the graphically for the cone shown in Fig. 2 (no. 32 in the 10 h_15 min variant):

Step I: $${u}_{1}=0.264\cdot {\mathrm{e }}^{\left(-0.38 \cdot {\tau }_{i}\right)}+0.107$$ ,$$\frac{d{u}_{1}}{d{\tau }_{1}}=-0.100\cdot {\mathrm{e }}^{(-0.38 \cdot {\tau }_{i})}$$

Step II: $${u}_{2}=0.372\cdot {\mathrm{e }}^{\left(-0.44 \cdot {\tau }_{i}\right)}+0.095$$ , $$\frac{d{u}_{1}}{d{\tau }_{1}}=-0.164\cdot {\mathrm{e }}^{(-0.44 \cdot {\tau }_{i})}$$

Step III: $${u}_{3}=0.397\cdot {\mathrm{e }}^{\left(-0.49 \cdot {\tau }_{i}\right)}+0.086$$ , $$\frac{d{u}_{1}}{d{\tau }_{1}}=-0.195\cdot {\mathrm{e }}^{(-0.49 \cdot {\tau }_{i})}$$

Step IV: $${u}_{4}=0.536\cdot {\mathrm{e }}^{\left(-0.44 \cdot {\tau }_{i}\right)}+0.080$$ , $$\frac{d{u}_{1}}{d{\tau }_{1}}=-0.236\cdot {\mathrm{e }}^{(-0.44 \cdot {\tau }_{i})}$$

Step V: $${u}_{5}=0.485\cdot {\mathrm{e }}^{\left(-0.46 \cdot {\tau }_{i}\right)}+0.076$$ , $$\frac{d{u}_{1}}{d{\tau }_{1}}=-0.223\cdot {\mathrm{e }}^{(-0.46 \cdot {\tau }_{i})}$$

Equations for changes (Fig. 3) in moisture content and extraction rate in consecutive extraction steps are also given for this cone (no. 17 in the 8 h_15 min variant):

Step I: $${u}_{1}=0.304\cdot {\mathrm{e }}^{\left(-0.53 \cdot {\tau }_{i}\right)}+0.113$$ ,$$\frac{d{u}_{1}}{d{\tau }_{1}}=-0.161\cdot {\mathrm{e }}^{(-0.53 \cdot {\tau }_{i})}$$

Step II: $${u}_{2}=0.292\cdot {\mathrm{e }}^{\left(-0.55 \cdot {\tau }_{i}\right)}+0.085$$ , $$\frac{d{u}_{1}}{d{\tau }_{1}}=-0.161\cdot {\mathrm{e }}^{(-0.55 \cdot {\tau }_{i})}$$

Step III: $${u}_{3}=0.369\cdot {\mathrm{e }}^{\left(-0.70 \cdot {\tau }_{i}\right)}+0.077$$ , $$\frac{d{u}_{1}}{d{\tau }_{1}}=-0.258\cdot {\mathrm{e }}^{(-0.70 \cdot {\tau }_{i})}$$

Step IV: $${u}_{4}=0.379\cdot {\mathrm{e }}^{\left(-0.71 \cdot {\tau }_{i}\right)}+0.059$$ , $$\frac{d{u}_{1}}{d{\tau }_{1}}=-0.269\cdot {\mathrm{e }}^{(-0.71 \cdot {\tau }_{i})}$$

Step V: $${u}_{5}=0.428\cdot {\mathrm{e }}^{\left(-0.77 \cdot {\tau }_{i}\right)}+0.060$$ , $$\frac{d{u}_{1}}{d{\tau }_{1}}=-0.330\cdot {\mathrm{e }}^{(-0.77 \cdot {\tau }_{i})}$$

Finally, equations for changes in moisture content and extraction rate in consecutive extraction steps are given for cone no. 5 in the 6 h_15 min variant:

Step I: $${u}_{1}=0.308\cdot {\mathrm{e }}^{\left(-0.58 \cdot {\tau }_{i}\right)}+0.0904$$ ,$$\frac{d{u}_{1}}{d{\tau }_{1}}=-0.179\cdot {\mathrm{e }}^{(-0.58 \cdot {\tau }_{i})}$$

Step II: $${u}_{2}=0.346\cdot {\mathrm{e }}^{\left(-0.63 \cdot {\tau }_{i}\right)}+0.1070$$ , $$\frac{d{u}_{1}}{d{\tau }_{1}}=-0.218\cdot {\mathrm{e }}^{(-0.63 \cdot {\tau }_{i})}$$

Step III: $${u}_{3}=0.368\cdot {\mathrm{e }}^{\left(-0.63 \cdot {\tau }_{i}\right)}+0.0837$$ , $$\frac{d{u}_{1}}{d{\tau }_{1}}=-0.232\cdot {\mathrm{e }}^{(-0.63 \cdot {\tau }_{i})}$$

Step IV: $${u}_{4}=0.387\cdot {\mathrm{e }}^{\left(-0.68 \cdot {\tau }_{i}\right)}+0.0838$$ , $$\frac{d{u}_{1}}{d{\tau }_{1}}=-0.263\cdot {\mathrm{e }}^{(-0.68 \cdot {\tau }_{i})}$$

Step V: $${u}_{5}=0.396\cdot {\mathrm{e }}^{\left(-0.65 \cdot {\tau }_{i}\right)}+0.0743$$ , $$\frac{d{u}_{1}}{d{\tau }_{1}}=-0.257\cdot {\mathrm{e }}^{(-0.65 \cdot {\tau }_{i})}$$

Figures 2a, 3a show the curves of actual changes in the moisture content of three sample cones subjected to different drying times (10 and 8 h) but the same immersion time (15 min); the curves were fitted to a model which is widely used in descriptions of drying at constant temperature (mostly for vegetables). The present study used variable temperature, which may have influenced the fit of the model, in addition to the input variables (drying and immersion times). The best fit was found for the cone subjected to the variant with 8 h of drying (Fig. 3), with a slight deviation in the first three extraction steps, and with a very good fit in the fourth and fifth steps. The lowest fit was found for the cone subjected to 6 h drying, which may be caused by insufficient drying time (the cone was exposed to 35 °C for 2 h, and to 50 °C for only 4 h).

Figures 2b, 3b show diagrams for cone extraction rates at different drying times (10 h and 8 h) at the same immersion times (15 min). As can be seen, extraction rates decreased in the very beginning, which is characteristic of the so-called second period of solid drying (Pabis^44^).

### Seed extraction dynamics

Table 2 presents data on the number of scales and seeds for the studied cones. There were from 33 to 70 open scales per cone, with an average of 48 (± 6). From 1 to 76 seeds were extracted per cone, with an average of 36 (± 18). Finally, each cone contained from 5 to 97 seeds, with an average of 52 (± 19). The weight of the extracted seeds ranged from 0.001 g to 0.651 g, on average 0.193 (± 0.109) g.

Cones obtained from different process variants did not differ in terms of the number of seeds extracted (*F* = 0.862 at *p* = 0.55) or their weight (*F* = 0.720 at *p* = 0.674). However, ANOVA did reveal significant differences in the number of scales (*F* = 3.561 at *p* ˂0.05) and the total number of seeds per cone (*F* = 2.93601 at *p* = 0.003645). Table 6 gives mean scale and seed numbers per larch cone (with standard deviations) for the various extraction variants and homogeneous groups.

**Table 6:** Mean numbers of cone scales and seeds for each process variant.

No	Variant	Number of scales *l*_*scales*_	Number of extracted seeds *l*_*n*_	Total number of seeds *l*_*w*_	Weight of extracted seeds *m*_*n*_ [g]
1	10 h_5 min	49^a,b^ ± 5	36^a^ ± 18	51^a,b^ ± 18	0.198^a^ ± 0.109
2	10 h_10 min	50^a^ ± 5	38^a^ ± 16	52^a,b^ ± 18	0.205^a^ ± 0.097
3	10 h_15 min	47^a,b^ ± 7	33^a^ ± 18	46^a^ ± 22	0.186^a^ ± 0.112
4	8 h_5 min	45^b^ ± 6	40^a^ ± 15	62^b^ ± 16	0.196^a^ ± 0.092
5	8 h_10 min	46^a,b^ ± 7	36^a^ ± 19	53^a,b^ ± 21	0.203^a^ ± 0.144
6	8 h_15 min	46^a,b^ ± 6	39^a^ ± 20	58^a,b^ ± 20	0.215^a^ ± 0.121
7	6 h_5 min	50^a^ ± 7	32^a^ ± 16	45^a^ ± 19	0.177^a^ ± 0.103
8	6 h_10 min	50^a,b^ ± 6	36^a^ ± 17	52^a,b^ ± 18	0.199^a^ ± 0.109
9	6 h_15 min	48^a,b^ ± 4	32^a^ ± 18	48^a^ ± 16	0.162^a^ ± 0.095

*a, b – homogeneous groups.*

On average, 70% of the seeds were extracted from cones used in all nine study variants, with 30% of the seeds remaining in the cones. Table 7 shows the number of seeds extracted in individual variants and the number of seeds remaining in the cones, expressed as a percentage.

**Table 7:** Number of seeds extracted from and remaining in the cones for each process variant.

No	Variant	Total number of seeds extracted [%]	Number of seeds remaining in the cones [%]	Number of seeds extracted in the chamber [%]	Number of seeds extracted in the drum [%]
1	10 h_5 min	72	28	66	34
2	10 h_10 min	73	27	69	31
3	10 h_15 min	72	28	64	36
4	8 h_5 min	65	35	61	39
5	8 h_10 min	67	33	65	35
6	8 h_15 min	67	33	58	42
7	6 h_5 min	71	29	61	39
8	6 h_10 min	70	30	60	40
9	6 h_15 min	67	33	56	44

The greatest number of seeds was obtained in process variants 2–73%, closely followed by variants 3, 1, and 7 (72%), and 8 (70%). The lowest seed yield was obtained from variant 4 (65%).

In all study variants, some of the seeds were obtained in the process of extraction in the chamber and some in the process of shaking in the drum (Table 7). The highest number of seeds in the chamber was obtained in variant 2 (69%), and the lowest in variant 9 (56%). On average, the largest quantity of seeds was obtained in the chamber in the 10 h variants, and the lowest quantity in the 6 h variants. Comparing different process variants of the same drying duration, the greatest number of seeds in the chamber were obtained in variants 2, 5, and 7 (and also in variant 8—only 1% fewer). The greatest quantity of seeds extracted by shaking in the drum was obtained in variant 9 (44%), and the lowest in variant 2 (31%). On average, 38% of seeds extracted in all variants were obtained by shaking in the drum.

**Figure 4:**
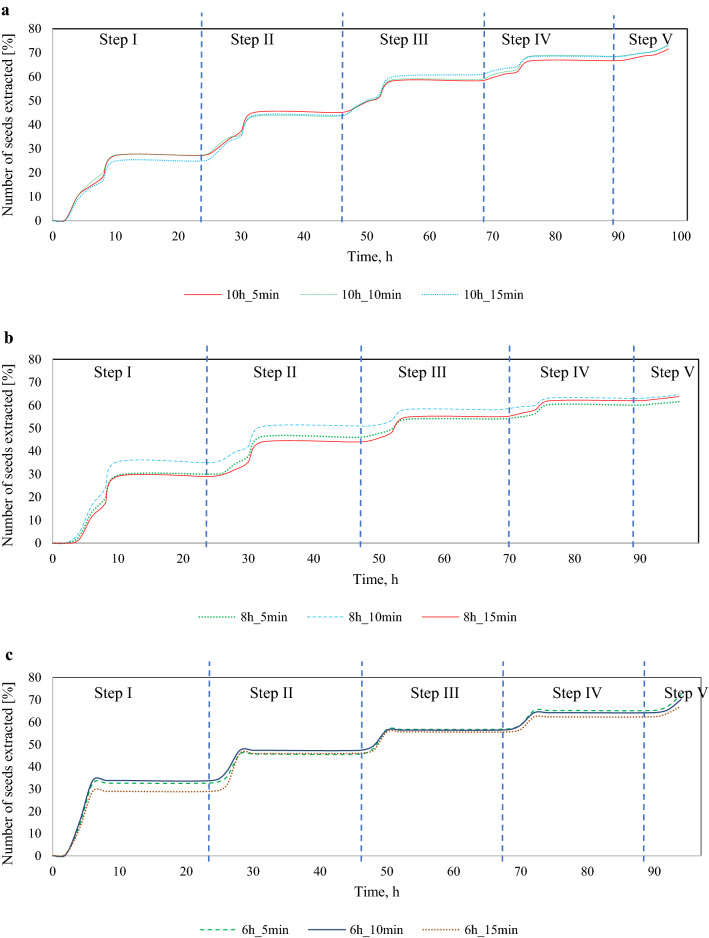
Percentage seed yield dynamics for each step of a five-step extraction process: (**a**) 10 h of drying, (b) 8 h of drying, (**c**) 6 h of drying.

It can be seen that in each of the variants and their individual steps, the highest number of seeds was obtained after 6 h of the process. Figure 4a–c shows the percentage of seeds obtained during the effective extraction time, where the number of seeds extracted at a given step was added cumulatively to those from the previous steps.

The diagrams in Fig. 4 show the percentage of seeds obtained throughout the entire process. Each step consists of drying, shaking, immersion, and soaking, except for step V, which involved only drying and shaking without immersion or soaking. Analysis of seed yield over 10 h of drying (Fig. 4a) shows that on average 37% of all extracted seeds were obtained in the first step, 26% in the second step, approx. 20% in the third step, 11% in the fourth step, and about 6% in the fifth step.

As regards the 8 h process (Fig. 4b), on average 30% of all extracted seeds were obtained in the in the first extraction step in the 8 h_5 min and 8 h_15 min variants, and as much as 53% in the 8 h_10 min variant. An average of 27% of all seeds were extracted in the second step, 15% in the third step, about 11% in the fourth step, and approx. 5% in the fifth step. The 8 h_10 min variant was characterized by the highest seed yield, beginning in the first step of the process (as compared to the 8 h_5 min and 8 h_15 min variants).

As far as the variant with 6 h of drying is concerned (Fig. 4c), on average approx. 46% of all extracted seeds were obtained in the first step, 24% in the second step, 15% in the third step, approx. 11% in the fourth step, about 4% in the fifth step.

When extracting seeds from larch cones, scale deflection and the number of obtained seeds are not assessed during the process, as is the case with pine and spruce cones due to the difficulties caused by the aforementioned morphology of larch cones (Tyszkiewicz, 1949). The presented diagrams show that a satisfactory seed yield (60%) was obtained in variants with 8 and 6 h of drying already after 10 h of effective extraction time.

The seed yield coefficient, α (3), and the cone mass yield coefficient, β (4), for each extraction variant are presented in Table 8.

**Table 8:** Seed yield coefficient and cone mass yield coefficient for each process variants.

No	Variant	Seed yield coefficient, *α*	Cone mass yield coefficient, *β*
1	10 h_5 min	0.71	0.050
2	10 h_10 min	0.73	0.052
3	10 h_15 min	0.72	0.049
4	8 h_5 min	0.65	0.045
5	8 h_10 min	0.68	0.053
6	8 h_15 min	0.67	0.048
7	6 h_5 min	0.71	0.041
8	6 h_10 min	0.69	0.050
9	6 h_15 min	0.67	0.038

The seed yield coefficient was the highest for variants 2 (0.73) and 3 (0.72), and the lowest for variants 4 (0.65) and 6 and 9 (0.67). The cone mass yield coefficient was the highest for variant 5, and the lowest for variant 9.

### Seed viability

Table 9 presents germination energy (*E*) and capacity (*Z*) for the control seeds as well as for seeds obtained from the various steps of the nine process variants, as well as their corresponding quality classes.

**Table 9:** Germination energy and capacity for the control seeds as well as for seeds obtained from the various extraction process variants.

No	Variant	Step I		Step II		Step III		Step IV		Step V		Average for the whole process		Quality class
		E	Z	E	Z	E	Z	E	Z	E	Z	E	Z	
C	Control sample	–										**45**	**57**	I
1	10 h_5 min	41	43	40	42	41	44	38	44	30	37	**38**	**42**	I
2	10 h_10 min	49	51	45	48	39	41	40	43	34	41	**41**	**45**	I
3	10 h_15 min	40	43	43	44	52	54	43	43	44	49	**47**	**47**	I
4	8 h_5 min	41	45	44	46	47	49	39	42	50	56	**44**	**48**	I
5	8 h_10 min	44	48	46	49	34	35	35	38	42	42	**40**	**42**	I
6	8 h_15 min	47	48	47	50	47	50	47	48	39	48	**43**	**47**	I
7	6 h_5 min	44	44	47	48	51	53	47	49	42	51	**46**	**49**	I
8	6 h_10 min	59	61	45	48	50	56	44	49	39	45	**47**	**52**	I
9	6 h_15 min	51	52	45	46	47	50	35	38	40	46	**43**	**46**	I

*E – energy, Z – germination capacity.*

Significant values are in bold.

Germination energy and capacity for the control sample were 45% and 57%, respectively, meaning that naturally released seeds, not subjected to any thermal or mechanical treatments, were classified in quality class I^18^. Importantly, seeds obtained from all the studied process variants were also placed in the same class; their germination energy ranged from 30 to 59%, and their germination capacity from 35 to 61%. When analyzing each extraction step separately, no correlation was found between decreasing germination energy and successive steps. However, the average germination energy was 46% for seeds obtained in the first extraction step of all nine variants, 45% for those from the second and third steps, 41% for seeds from the fourth step and 40% for those from the fifth one. Thus, in each subsequent step the average germination energy of seeds was equal or lower than in the previous step, which is consistent with literature reports that prolonged drying may reduce the quality of seeds^8^. This is also corroborated by the fact that the highest germination energy and capacity was revealed by seeds from variants with 6 h of drying while the lowest germination indicators characterized seeds from the 10 h variants. Furthermore, seeds from variant 1 exhibited the lowest germination energy and capacity and seeds from variant 8–the highest.

Another reason for the higher quality of seeds from variants with 6 h of drying may be the lower initial moisture content of the cones due to the longer time they were kept at room temperature immediately before the test (u_01_ = 0.391 ${\mathrm{kg}}_{\mathrm{water}}\cdot {\mathrm{kg}}_{d.\mathrm{w}.}^{-1}$ as compared to u_01_ = 0.411 ${\mathrm{kg}}_{\mathrm{water}}\cdot {\mathrm{kg}}_{d.\mathrm{w}.}^{-1}$ for seeds from variants with 8 and 10 h of drying). These results are in line with the study of Tyszkiewicz^8^, who noted that under the same temperature and humidity conditions, the quality of seeds from cones with a lower moisture content did not deteriorate, in contrast to the quality of seeds obtained from cones with a higher moisture content.

The germination capacity of seeds calculated from the mean capacity of seeds obtained from the same extraction steps of all process variants was similar at 45% for each of the steps.

In summary, in the study the authors investigated a five-step process of extracting seeds from larch cones involving immersion and heat treatment to maximize seed yield. It was found that the two-step process widely used in extractories is insufficient, while a four-step process does not lead to a significantly higher number of obtained seeds. Thus, a three-step process appears to be optimal.

## Discussion

The height and diameter of the cones used in the study were within the limits described by other researchers, i.e., a height of 4 cm and a diameter of 2 cm^8^, or a height of 2–5 cm^47^ or 4–5 cm^48^. Since climatic factors (annual air temperature and precipitation) affect the production of, e.g. pine cones to a greater extent than non-climatic ones^25^, further research on these effects should also be conducted for larch. The high initial moisture content of the studied cones could have been caused by the absence of preliminary drying, as is the case in practice in seed extraction facilities, as well as by the early date of cone harvesting (December)^8^.

Multiple drying and immersion increased the initial moisture content and decrease the final moisture content in each successive extraction step. Appropriate drying and immersion procedures (rehydration) in the studied process variants enabled intensive water absorption by the tissues of the material being dried, leading to greater weight and volume^49^. Some studies have reported the effects of changes in the rheological properties of resin particles between cone scales during extraction. At approx. 50 °C resin is sufficiently “liquefied” (melted) to enable scale opening in pine cones (in the force range of 4.7–10 N). In turn, at constant stress levels, as is the case with the movement of individual scales, resin gradually begins to “flow” (it turns less viscous) as temperature increases over time. Consequently, the forces needed to overcome the adhesive force of resin decrease (1.99 N), and it retreats cohesively. According to the literature, high temperatures (~ 50 °C) facilitate cone opening in *P. sylvestris*, while high moisture content does not affect the rheological properties of resin as it is completely insoluble in water^50^.

Cones subjected to 8 h of drying exhibited an average moisture content of 7.1% after the fourth extraction step and 6.4% after the fifth step. This means that those cones maintained a downward trend in moisture content as opposed to cones subjected to 10 h of drying, which had a similar level (approx. 7%) of moisture in the fourth and fifth extraction steps of the process. This shows that the optimal duration of drying is 8 h, which is in line with the techniques used in practice^3^.

Seeds may be extracted from larch cones using either a thermal process with immersion or a thermo-mechanical one. While both processes are used by extractories, they are not standardized or well-studied. The thermal method is less efficient than the one involving scale crushing and abrasion. On the other hand, larch seeds obtained by thermal extraction are characterized by somewhat greater germination capacity, lesser damage of cell membranes, and greater resilience in accelerated ageing tests, as compared to seeds extracted by mechanical crushing; furthermore, the former are easier to clean^3^.

The highest seed yields were recorded in the 10 h_10 min and 10 h_15 min variants (73%). A similar seed yield was also obtained in the 6 h_5 min process (72%), which shows that the extraction process can be shortened and immersion time can be reduced without significantly compromising the quantity of obtained seeds. The lowest seed yield in the drying chamber was obtained in the 6 h_15 min variant (56%) due to insufficient drying time and excessive immersion time. A failure to sufficiently reduce the moisture content of cones during the extraction process prevents seed movement within the scales^18^. For a long time now researchers have been studying phenomena involving wood that can adopt a deformed shape at a certain moisture content and then return to its original shape after absorbing water^51–53^ as well as scale movements in conifer cones caused by their cellular structure^54–59^. Findings from such research can form the basis for analyzing multi-phase movement by artificial intelligence^60,61^.

Each successive extraction step resulted in seeds with lower mean germination energy, but germination capacity remained at a similar level in all steps, which means that the obtained seeds belonged in quality class I.

In the first three steps of the extraction variant with 8 h of drying the seed yield was approx. 59%, as compared to an average of 8% in the fourth step, and approx. 4% in the fifth step. Therefore, this variant appears superior among the nine studied. In the first three steps of the extraction variant with 10 h of drying, the seed yield was approx. 57%, in contrast to approx. 9% in the fourth step, and approx. 5% in the fifth one. Finally, in the first three steps of the extraction variant with 6 h of drying, the seed yield was approx. 56%, as compared to an average of 9% in the fourth step and approx. 5% in the fifth step. As can be seen, the minimum number of extraction steps needed to obtain an approx. 60% seed yield is three, which is consistent with reports by other authors^3,40^.

An analysis of the value of European larch seeds should be based on their origin and the costs of extraction energy. First, attention should be given to the provenance of larch cones and their genetic (e.g., species) purity. Second, if the seeds are extracted for the purpose of long-term storage, the right extraction conditions are critical to avoid compromising seed quality due to excessive drying and immersion time. Third, the economic rationale of seed extraction should be considered given the high energy costs to address the question whether it would be possible to use a shorter drying time (6 h) with immersion (5 min) and a minimum of 30 min of shaking after each step, which was shown to result in a seed yield of 71% in the present study.

The power rating of the laboratory dryer used in this study was 2.2 kW, which means that energy consumption per drying step was 22, 17.6, and 13.2 kWh for variants with 10, 8 and 6 h of drying, respectively. Consequently, energy consumption in a five-step process was 110, 88, and 66 kWh, accordingly. It should be noted that the difference between the longest and the shortest drying time was significant. Energy consumption for an extraction process involving three drying steps of 8 h each would be 52.8 kWh, which is twice as low as for a process with five drying steps of 10 h each.

In the case of dryers used in Polish seed extraction plants, their installed capacity is about 25 kW. The results of the estimations carried out for laboratory tests indicate the scale of possible energy savings if the improved cone extraction processes proposed in this publication is used in industrial settings. For the purposes of technical and economic evaluation involving energy consumption, further research is needed with more detailed measurements not only of the drying step (carried out in dryer - stage (a) on the figure 1), but also of the shaking step in the drum (stage (b) on the figure 1).

## Conclusions

1. A comparison of nine variants of five-step seed extraction revealed the highest yield for the 10 h_10 min variant (73%). In the first and second steps, the highest mean yields were obtained for the 8 h_10 min variant (53% and 32%, respectively), while in the third step the highest yield was found for the 10 h_5 min (22%) variant. In the fourth step, all variants exhibited similar yields, of approx. 11% Finally, in the fifth step, seed yield increased with drying duration (on average 5% for 10 h and 8 h and 4% for 6 h).
2. The recommended process consists of three 8 h drying steps and two (rather than five) 10 min immersion steps, as it led to a yield of 59%, which was greater than in the other variants. The gains from subsequent extraction in the next two steps were very low (approx. 11% and 5% of seeds in the fourth and fifth steps, respectively). A five-step process may be conducted when the cones are derived from a valuable source and it is advisable to extract the highest possible quantity of seeds.
3. The mean germination energy and capacity did not differ significantly between the selected process variants. The mean results exceeded 40%, which places the extracted seeds in quality class I. Extended drying times does not significantly lead to decreased seed quality.
4. The extraction process can be described according to Lewis’s empirical model for the second drying stage. In those equations, the initial moisture content of cones (u_01_) usually increased with each successive process step due to immersion. Furthermore, in each step cones exposed to the longest immersion time (15 min) were characterized by a much higher moisture content than the ones exposed to shorter immersion times. The same was true of the final moisture content, with the highest values found for cones following 15 min of immersion, and the lowest for cones after 5 min of immersion. The *b* coefficient ranged from 0.34 to 0.60; it was noted that in the 8 h_15 min variant it increased with each successive step.

## List of symbols

*u*: Instantaneous cone moisture content during seed extraction ${[\mathrm{kg}}_{\mathrm{water}}\cdot {\mathrm{kg}}_{\mathrm{d}.\mathrm{w}.}^{-1}]$,
*u*_*01-5*_: Initial cone moisture content in a given extraction step (1–5) ${[\mathrm{kg}}_{\mathrm{water}}\cdot {\mathrm{kg}}_{\mathrm{d}.\mathrm{w}.}^{-1}]$,
*uk1-5*: Final cone moisture content in a given extraction step (1–5) ${[\mathrm{kg}}_{\mathrm{water}}\cdot {\mathrm{kg}}_{\mathrm{d}.\mathrm{w}.}^{-1}]$
*b1-5*: Coefficient characterizing moisture content change in a given step (1–5) [h–1]
$$\uptau$$_i_: Time [h]
*e*: The base of the natural logarithm
*α*: Seed yield coefficient
*l*_*n*_: Number of extracted seeds
*l*_*w*_: Total number of seeds
*β*: Cone mass yield coefficient
*m*_*n*_: Weight of extracted seeds [g]
*m*_*0*_: Initial cone weight [g]
*h*: Height [mm]
*d*: Diameter [mm]
*W*: Cone moisture content [%]
*l*_*scales*_: Number of scales
*E*: Energy
*Z*: Germination capacity
*R*_*cr*_: Critical value of the correlation coefficient
